# “Comorbidity” between epilepsy and headache/migraine: the other side of the same coin!

**DOI:** 10.1007/s10194-011-0371-z

**Published:** 2011-07-31

**Authors:** Pasquale Striano, Vincenzo Belcastro, Alberto Verrotti, Pasquale Parisi

**Affiliations:** 1 Muscular and Neurodegenerative Diseases Unit, ‘G. Gaslini’ Institute, University of Genova, Genoa, Italy; 2 Neurology Clinic, S. Anna Hospital, Como, Italy; 3 Chair of Paediatrics, University of Chieti, Chieti, Italy; 4 Child Neurology, Chair of Paediatrics, II Faculty of Medicine, ‘La Sapienza’ University, Rome, Italy

Dear Editor,

We read with interest the paper by Toldo and co-workers addressing the important issue of comorbidity between epilepsy and headache/migraine [1]. In fact, although these are both chronic disorders with episodic attacks and their association has been long recognized, the common molecular mechanisms remain so far elusive [2–4]. Indeed, recent data suggest shared genetic substrates and phenotypic-genotypic correlations with mutations in some ion transporters genes, including *CACNA1A, ATP1A2, SCN1A* [3, 4]. In their latest study, Toldo et al. evaluated the distribution of five polymorphisms of *SCN1A*, the gene encoding the a-subunit of the neuronal voltage-gated sodium channel Nav1.1, in children and adolescents with headache and epilepsy compared to controls. They concluded that SCN1A is not involved in the pathogenesis of comorbidity between headache/migraine and epilepsy [1].

Despite this negative study, neuronal hyperexcitability and increased susceptibility to cortical spreading depression remain important molecular mechanisms in the pathophysiology of this association. In the coming decade it is possible that International efforts to collect large well-phenotyped samples and the current technical possibilities of massive genotyping will shed light on the genetic mechanisms involved in with headache/migraine and epilepsy. On the other hand, the role of additional, non genetic factors influencing the excitation threshold, e.g., mitochondrial dysfunction, disturbance in neurotransmitters metabolism, or inflammatory factors-alone or in combination-can not be excluded a priori [3, 4]. Indeed, any of these triggering factors, irrespective of their nature (genetically determined or not), could potentially lead to a paroxysmal and transient cortical excitability change leading to prolonged neuronal depolarization (seizure) or spreading depression (headache/migraine) [2, 3].

Among the potential practical implications arising from these observations, there is the urgent need for a revision of either International Classifications of Epilepsy and Headache disorders [2, 3, 5]. Finally, insight into the molecular mechanisms involved in the association between headache/migraine and epilepsy is crucial to identify drug targets for improving patients’ treatment.
